# Letter to the Editor: Feasibility of an alternative, physiologic, individualized open-lung approach to high-frequency oscillatory ventilation in children

**DOI:** 10.1186/s13613-020-00695-3

**Published:** 2020-06-08

**Authors:** Arpita Chattopadhyay

**Affiliations:** Pediatric Intensive Care Unit, Department of Pediatrics, Chacha Nehru Bal Chikitsalaya, Geeta Colony, New Delhi, 110031 India

We read with great interest the article by Jager et al. [1]. The authors should be commended for highlighting a step-by-step strategy of recruitment of lungs in PARDS patient on HFOV, early in the course of illness rather than as a rescue strategy. The authors set a high frequency (12 Hz) and amplitude (70–90 cm H_2_O) in all patients with PARDS, rather than the conventional values based on patient’s age or weight and chest wiggle, and then followed a stepwise mean airway pressure (mPaw) titration to determine the lowest mPaw needed (2 cm H_2_O above mPaw_derecruitment_) to ventilate the lungs [1]. By doing so, they hypothesize oscillating the patient on the deflation limb of the P–V loop. Setting a high frequency allows for very small tidal volumes to be delivered, also allowing for more efficient opening of collapsed lung regions [2].

Re-exploring the concept of corner frequency (Fc), previously described by Venegas et al. [3] about a decade ago, Fc = 1/(2*πRC*), where *R* is resistance and *C* compliance. They defined Fc as the frequency on HFOV, at which optimal gas exchange occurs in combination with the least injurious pressures. Fc is dependent on the underlying lung pathology—it is increased in lung diseases with short time constants and low compliance, such as PARDS. This implies that at higher F, alveoli are ventilated at minimal pressure required for ventilation, as opposed to lung diseases characterized by prolonged time constants, such as asthma or bronchiolitis [4]. As reported in previous studies [5, 6], high-frequency ventilation as a ventilation strategy is more efficient in certain PARDS types—such as airleak syndromes, inhalation injuries, secretion induced lung collapse. It would be worth sharing data regarding the group of patients of PARDS who responded best to the recruitment manoeuvre: whether it was ones with homogenous PARDS phenotype versus those with heterogenous lung pathology. Overdistension of healthy lungs in heterogenous lung disease precludes the benefits achieved by high mean airway strategy in HFOV. The authors may elaborate on these aspects to give an idea about how the recruitment strategy was tolerated across different etiologies of ARDS.

The volume of gas generated by each frequency wave is highly variable and depends on circuit tubing, humidifier, endotracheal tube diameter and length. Even in the current study, the authors set out at frequencies of 12 Hz for mild/moderate PARDS and dropped to a frequency of 10–10.5 Hz within the first 6 h of the recruitment manoeuvre, the tolerability of high frequency irrespective of weight and age could be explained, as suggested in the table of baseline characteristics—that children between 0 and 12 months comprised 75%, 69% and 66.7% of study population, for mild, moderate and severe PARDS, respectively. Even traditionally, the set frequency at this age/weight ranges between 10 and 12 Hz [7]. It will be worthwhile sharing the outcomes by the age groups, even though the numbers in children beyond infancy are small. It may be inappropriate to suggest this strategy of using high ‘F’ in older children without adequate data.

It would have been interesting to provide figures regarding the proportion of all PARDS (by severity) who were transitioned to HFOV as per protocol. The Conventional Mechanical Ventilation (CMV) settings prior to initiation of HFOV are quite similar in the three categories of PARDS, except for FiO_2_. It appears that there may have been a suboptimal use of CMV as the patients with severe PARDS had high FiO_2_ requirements [median 0.99 IQR (0.75–1.00)] prior to recruitment manoeuvre and transition to HFOV, yet the median PEEP was 7.4 (6.7–9.9) cm H_2_O despite an oxygenation index of 38 (median). It is not clear if this was part of the protocolized management in the unit. One would anticipate most patients to be treated with higher PEEP (> 10 cm H_2_O) at this stage, unless the unit protocol had restrictions on high PEEP. Eventually it is often difficult to manage mechanical ventilation by the rule book and decisions are best taken on a case-to-case basis as per discretion of the treating physician. It is also worthwhile noting that patients were not proned.

In this era where non-invasive modes of ventilation are gaining momentum, perhaps patients with mild PARDS should have been excluded from this strategy, although they mention no increase in barotrauma or increased vasopressor requirements with such settings.

We hope this study paves the way forward for the design of future clinical studies or randomized trials for the optimal strategy to use HFOV.

## Data Availability

None.
